# What are the perceived added values and barriers of regulating long-term care in the home environment using a care network perspective: a qualitative study

**DOI:** 10.1186/s12913-018-3770-x

**Published:** 2018-12-06

**Authors:** Didi Verver, Annemiek Stoopendaal, Hanneke Merten, Paul Robben, Cordula Wagner

**Affiliations:** 1 Department of Public and Occupational Health, Amsterdam UMC, Vrije Universiteit Amsterdam, Amsterdam Public Health research institute, Van der Boechorststraat 7, NL, 1081 Amsterdam, BT Netherlands; 2 Erasmus School of Health Policy and Management (ESHPM), Erasmus University Rotterdam, Burgemeester Oudlaan 50, 3062 Rotterdam, PA Netherlands; 3 Netherlands Institute for Health Services Research (NIVEL), Otterstraat 118-124, 3513 CR Utrecht, the Netherlands

**Keywords:** Government regulation, Long-term care, Community network, Independent living

## Abstract

**Background:**

Changes in Dutch policy towards long-term care led to the Dutch Health and Youth Care Inspectorate testing a regulatory framework focusing on care networks around older adults living independently. This regulatory activity involved all care providers and the older adults themselves.

**Methods:**

Semi-structured interviews with the older adults, and focus groups with care providers and inspectors were used to assess the perceived added value of, and barriers to the framework.

**Results:**

The positive elements of this framework were the involvement of the older adults in the regulatory activity, the focus of the framework on care networks and the open character of the conversations with the inspectors. However, applying the framework requires a substantial investment of time. Care providers often did not perceive themselves as being part of a care network around one person and they expressed concerns about financial and privacy issues when thinking in terms of care networks.

**Conclusions:**

The experiences of the client were seen as important in regulating long-term care. Regulating care networks as a whole puts cooperation between care providers involved around one person on the agenda. However, barriers for this form of regulation were also perceived and, therefore, careful consideration when and how to regulate care networks is recommended.

## Background

### The increasing number of frail older adults living independently and their use of care

Older adults often have a variety of chronic conditions [1] for which they usually need the services and long-term care of multiple care professionals and organizations [2, 3]. According to the European Commission and the European Union’s advisory body ‘the Social Protection Committee’ [4], long-term care is defined as “a range of services and assistance for people who, as a result of mental and/or physical frailty and/or disability over an extended period of time, depend on help with daily living activities and/or are in need of some permanent nursing care”. The multiplicity of the care need may lead to care that is complex and fragmented [5, 6]. Continuity of care and personal relationships between the care provider and client are determinants of the quality of care [7, 8]. Hence, having multiple different health care providers is identified as a specific indicator of risk within long-term care for the elderly [9]. The continuity of care is even more relevant for frail older adults, who have an accumulation of physical, psychological and/or social problems [10] and thus usually have to deal with different care providers.

### Dutch healthcare system

Since January 2015, the Dutch government changed the organization and financing of its long-term care system. Currently, the Dutch healthcare system is controlled by four principal laws; the Dutch Health Insurance Act (Zvw), the Long Term Care Act (Wlz), the Social Support Act (Wmo) and the Youth Care Act (Jeugdwet) providing a modest role for the government. It has as social insurance background and a dominant role for healthcare insurers [11]. With these changes, policy is now even more focused on enhancing self-sufficiency, independent living and informal care. Furthermore, local government has increasingly borne the burden of responsibilities regarding the care and support for their populations living independently. More care will be delivered at home since long-term care users are expected, by law, to stay independent as long as possible. Residential care is only possible for those who need intensive care and 24 h supervision [12, 13]. The numbers of older adults are rising in many countries, including the Netherlands [10, 14]. This means that it becomes more common for different care providers, formal as well as informal, to deliver complex care to the same client in the home setting. Care for older adults is generally seen to be provided by an extensive network comprising institutions, professionals, technologies, relatives, friends, and other caregivers [15, 16]. This makes it difficult for care providers to coordinate care and support as it is often unclear which care provider takes responsibility for these [5]. However, cooperation and exchange of information is needed between professionals, and between professionals and informal carers, in order to provide optimal care to the client [17]. In this study we follow the definition of ‘informal care’ as described by the Dutch Ministry of Health, Welfare and Sport (VWS): long-term care for those in need, by people in their direct social network and on the basis of a social relationship, and not provided on a professional basis [18]. However, in this study we additionally chose to include welfare providers and volunteers among informal care providers.

### Health care regulation

The Dutch Health and Youth Care Inspectorate (IGJ) guards the quality and safety of care, enforcing laws, scientific guidelines and field standards [19]. The IGJ supervises and promotes quality and safety of care and is part of the Dutch Ministry of Health, Welfare and Sports. The IGJ has two main methods to regulate care; incident based and risk based. With the first the IGJ responds to notifications about low quality of care by citizens, care providers or others. With risk based regulation the IGJ proactively regulates care where the highest risks are expected. When the IGJ identifies low quality of care, or unstable situations in the care process, they are able to intervene and enforce actions. These actions vary from advice up to criminal justice [20].

There are many quality standards and guidelines for care institutions and individual care professionals which can be used for the regulation of residential care. Since more care will be delivered in the home setting and the rising number of care providers involved, regulation in the home care environment becomes even more important and complex. Firstly, there are no specific scientific guidelines nor quality and safety norms for regulation of care networks [21, 22]. The IGJ speaks of a care network when multiple care providers, formal and informal, and organizations are involved in long-term care for frail people living independently [23]. Secondly, home care is usually provided by multiple care providers and care organizations. This means that the IGJ does not have one responsible authority to address for the regulation of home care. Thirdly, the lack of agreements about cooperation within the network makes it difficult for a health care inspector to address problems with the quality or safety of care. A fourth complicating factor is that not all the participants within the care network fall under the regulatory regime of the IGJ. These participants, for example, may also include informal care providers or care and support organized by the local governments. However, communication and cooperation between the formal and informal care providers is of utmost importance and they are therefore seen as part of the total care network by the IGJ.

Changes in care providers involved in a client’s care may hinder the continuity of care [24]. Quality of care is therefore often negatively influenced by the involvement of multiple different health care providers [9]. To address these issues, the IGJ developed and tested a regulatory framework for care networks in long-term care at home [23]. There is an increasing focus on public participation in regulation in different European countries [25–28]. Inspectorates aim to make more active use of information gathered from clients as sources of information for the regulatory process. Potentially, they may provide relevant signals about the quality of care on ‘softer’ aspects, but also on technical issues of quality [29]. It is, however, unclear what the best ways are for involving clients in health care regulation, especially in the home environment. With this in mind, the main aim of the new regulatory framework developed by the IGJ was to start the regulatory activity by interviewing the older adult and to describe and assess the care network from their perspective.

### Aim and objectives

In 2014, a project group was formed consisting of seven inspectors of the IGJ with different expertise within the domain of long-term care. The inspectors of this project group had the assignment to develop and test a new regulatory framework, aiming to improve the regulation of care networks of professionals and organizations in long-term care provided in the home environment [23]. The perspective of the client should be represented in this regulation activity and it should fit the client’s needs and experiences. To our knowledge, this way of regulating long-term care is not used or tested in any other country. The results might be useful for all types of organizations aiming at regulating or supervising care network activities.

The new regulatory framework was targeted at three vulnerable population groups who received care at home. These were adults with a mental disability, frail older adults and adults with chronic psychiatric disorders. This paper only focuses on the frail older adults, since the study described in this paper was part of a larger study which focuses on frail older adults [30]. The new regulatory framework was analyzed from three different perspectives: the older adults; their care providers; and the inspectors. This was done to assess whether the information needed to regulate the care networks could be collected with this framework. This study focused on both the added value to, and the barriers experienced with the use of the framework. Research concerning care networks is a relatively new area [21, 31] especially regarding regulatory activities [32]. This research will contribute to knowledge in this field.

## Methods

Firstly, the description of the content of the framework and the process of the regulatory activity are explained in order to support the replication of our study by other countries and provide background information about the steps that were taken by the inspectors and later on analyzed by the researchers. Secondly, the methods used for the analysis of the regulatory activity are described.

### Regulatory activity

The IGJ indicated four themes essential to the network of care delivered to frail older adults. These were: the client as the point of focus; integrated and coordinated care; informal care and/or volunteers; and safety. These four themes were based on a risk assessment and were underpinned with existing ‘field norms’. These field norms include quality frameworks, professional standards and quality indicators for responsible care, set by healthcare professionals. The IGJ tested their new regulatory framework in two regions in the Netherlands from April to December 2015. The use of the framework was experimental and there were no consequences for the professional care providers, unless serious dysfunctioning was established. This facilitated care providers who participated into providing full disclosure.

The following steps were taken by the IGJ to test their framework:

1. Using the four themes, inspectors spoke with clients and, when present, their informal care provider about their care network and their experiences with the care they received. They recruited the clients via different organizations in the two regions, one urban area and one rural area. The older adults had to experience somatic or psychogeriatric problems. Also, professionals of the organizations had to indicate them as frail according to their own opinion. Furthermore, they had to be willing to participate. When clients decided to participate they signed an informed consent form and the IGJ visited the respondents at their own home.
2. After informed consent was given by the clients during the conversation, the inspectors spoke with the care providers who were most involved up to a maximum of five, formal and informal, in the care network. They asked the care providers about the functioning of the care network using the field norms belonging to the four themes. Some examples of questions were: ‘does the client receive support in order to formulate his needs for care delivered at home?’; ‘are care and welfare delivered at home integrated in dialogue with the client?’; ‘ can informal care providers make sure that formal care providers in the care network take into account the wishes of, and options available through, the informal care provider and adjust formal care to these options?’.
3. The conversations were recorded and analyzed by the inspectors using ATLAS.ti version 7 [33]. The results relating to the functioning of the network were interpreted per network and at the regional level.
4. The findings relating to the functioning of the care networks per region were presented during a meeting with the care providers in each region. Written results were also handed to the care providers who were present during those meetings. The rationale behind this was to give feedback to the care providers about the results of the regulatory activity, the number of clients involved and their care providers and the functioning of the care networks based on the four themes i.e. the client as the point of focus; integrated and coordinated care; informal care and/or volunteers; and safety.

The regulatory activity of the IGJ was analyzed to assess whether the information required to assess the functioning of a care network in the home situation could be collected with this framework. Because of the exploratory nature of this study and the lack of previous research it was chosen to perform the research using multiple qualitative methods [34]. The aim was to gain insight into the experiences of the three types of respondents - clients, care providers, and inspectors - in relation to the regulatory framework and to assess whether the respondents perceived the framework as valuable for regulating care. This study was part of a larger study [30], which was embedded within the “Academic Collaborative Center on Supervision” in Dutch the Academische Werkplaats Toezicht, or AWT, established in 2011. In order to expand scientific knowledge about health care regulation, different research institutions work together with the IGJ within the AWT.

The larger study was approved by the Scientific Committee of the EMGO+ institute (WC2013–002) and the Medical Ethical review committee of the VU University Medical Center Amsterdam (2013/216). All information gathered about the participants was used only for this study and was processed separately from participant identifiers in order to protect their privacy and confidentiality.

### Data collection

Different research methods were used to collect data. These were semi-structured interviews with older adults, two focus groups with care providers in the two participating regions, a focus group with the inspectors evaluating the regulatory framework and an analysis of the inspectors’ logbooks. IGJ inspectors asked the older adults and/or their informal care provider during their conversation whether they were willing to share their experiences relating to the regulatory activity with the researchers. The inspectors also asked clients for their informed consent to participate in the research project. When the older adults gave informed consent the IGJ sent the informed consent forms to the researchers so they could contact the older adult or their informal care provider. The researchers contacted them by phone to make an appointment for the semi-structured interview. It was left up to the older adults or informal care providers whether they wanted to do the interview by phone or face-to-face in their own home.

Six interviews by phone were audio recorded and written on paper during the interview, three face to face interviews were audio recorded and written on paper during the interview, two interviews by phone were only summarized on paper and afterwards member checked by the respondents and one interview by phone was summarized on paper. Research has shown that telephone interviews can be used productively in qualitative research [35]. In this study non-verbal communication was not a primary interest for the researchers, interviews by phone were, therefore, a suitable replacement for face-to-face interviews. All interviews were held by one female PhD student, DV, who is experienced in interviewing older adults. Prior to the interview DV explained the aim of the interviews, her own background as a health scientist and the confidentiality of the information that the respondents will give. Background information, i.e. age, gender and living situation, was already gathered by the inspectors, which the researchers had access to after written informed consent by the respondents, so this information did not had to be asked again. The semi-structured interview contained topics about: reasons to participate in the regulatory activity; their perceived importance of the involvement of older adults in regulation; experiences with the conversation with the inspectors; their time investment and frailty. Frailty was measured in the interview using a quantitative questionnaire, the Tilburg Frailty Indicator (TFI). This was done to verify whether the older adults who participated were indeed frail [36]. The TFI is an instrument which uses 15 dichotomous items concerning frailty on three different domains; physical, psychological and social. A score of five or higher was classified as being frail. The developers of this questionnaire assessed the internal consistency as Cronbach’s α = 0.73, indicating moderate internal consistency [37]. The TFI currently has robust evidence of reliability and validity and its psychometric properties have been most thoroughly tested according to recent research [38].

The interview guideline was developed in close collaboration with the project group of the IGJ. Firstly, the project group pointed out what information they found important to evaluate their regulatory activity. Secondly, the researchers included these topics into a proposal for the evaluation, including the pre-defined research questions. Thirdly, the project group commented on this proposal. As a fourth step, topics were distilled from these research questions. These topics again were commented on by the project group.

Two focus groups were organized for the professional care providers involved in the regulatory activity, one in each region where the IGJ tested their regulatory framework. The aim of these focus groups was to assess the experiences of the care providers with the regulatory framework. They took place immediately after the meetings organized by the IGJ for the care providers in November and December 2015. Together with the invitation for the meetings, the IGJ asked the care providers to participate in the focus group. In region one, eight out of the 30 care providers participated and in region two, five out of the 33 care providers participated. During the meeting with the IGJ, the care providers received the results about the functioning of the care networks and the recommendations of the IGJ. During the focus groups the care providers gave information to the researchers about their experiences with, and opinions concerning, the regulatory framework and the contact they had with the inspectors. The inspectors were not present during these focus groups. Prior to the focus group, the project group gave the researchers insight into their findings and results, so the researchers were able to select topics for the focus groups with the care providers. The following list of topics was discussed during the focus groups: bottlenecks for the framework; missing subjects; recognizable results; the added value of using the client as a starting point; and the possible consequences of the framework.

The following step in this study was establishing a focus group with the inspectors, the members of the project group, involved in the development and implementation of the framework. The aim of this focus group was to obtain information about the experiences of the inspectors using the framework. The list of topics which was discussed during this focus group contained the following: the added value of involving the client; the different target groups; loss of control of the clients’ lives; information gathered through the framework; and the potential effects of the framework.

During all the focus groups there was one moderator (HM) and one minutes secretary present (DV). HM, female PhD, is experienced in moderating focus groups as a researcher and a teacher. The moderator introduced the topics of the list of subjects and made sure all respondents were heard. The minutes secretary took minutes during the focus groups, tracked time in order to make sure all topics were discussed and recorded the meetings.

As a last step, the researchers analyzed the logbooks of the inspectors involved. The inspectors all kept track of the time they invested in the project. The researchers were then able to analyze the time spent by the inspectors on capturing the functioning of the network around one individual older adult.

### Analysis

The answers to the semi-structured interviews were digitalized in Microsoft Excel. Inductive content analysis was performed [39]. Answers to open-ended questions were coded and categorized by one researcher. A second researcher checked the topics and key-aspects that emerged from that process. In the case of disagreement, both researchers discussed the specific answer and came to an agreement about the topic and key-aspects.

The minutes were used as data to analyze the focus groups. Inductive content analysis was performed and the results were summarized. These summaries were sent per email to all members for them to verify our findings. Suggestions made by the respondents on the summaries were added. The focus groups were recorded and stored as audio files, together with the minutes and the summaries, with feedback from the focus group members.

## Results

We first provide some characteristics of the older adults who participated and how they experienced their involvement in the regulatory process. We then elaborate on the perspective of the care providers, followed by the perspective of the inspectors, on the potential of, and barriers to, this regulatory framework.

### Older adults

The researchers interviewed 12 out of the 15 older adults or their informal care provider who were involved in the regulation activity. Three respondents were not interviewed by the researchers either because they indicated that the burden was too high for them or they did not want to participate. In seven of the 12 cases the interviews were held with the older adult alone and in five cases the informal care provider of the older adult was also present. Four interviews were conducted face-to-face and eight by telephone. The mean age of the older adults was 82,45 (SD: 9,4), see Table 1 for the distribution of age and gender.

**Table 1:** Distribution of ages

Age	64	70	85	90	74	80	83	89	90	90	92	–
Gender	m	m	m	m	f	f	f	f	f	f	f	f

The majority of the respondents said that the conversation with the inspector was enjoyable and it was not experienced as a burden. The inspectors were friendly, they understood their situation well, listened carefully and invited them to give honest answers. A criticism voiced by one respondent was that the conversation with the inspectors took quite a long time, approximately 60 min. Another respondent mentioned that the conversation was emotional. Also, one respondent mentioned that it seemed like the inspectors were searching for the right way to apply the regulatory framework. This was expressed as: OAT01“Oh well, it seemed they were searching,(asking) ‘what do we need to assess the total image’. I thought they just started it, I was one of the first they approached”.

All the respondents expressed that they were able to provide the inspectors with a clear image of the care providers involved and thus of their care network. All respondents indicated that involving the client for regulation purposes was important: OA02 “Then they [inspectors] know how it goes, otherwise they will never know”. Eight respondents indicated that the situation of the older adults was crucial in this matter. They mentioned that their condition has to be serious, but they still have to be capable of answering the questions asked by the inspectors or, if not, by an informal care provider instead.

The older adults mapped out their care network and gave the names of their care providers involved to the inspectors. Seven of the 12 respondents did not see any problems in involving the professional care providers in the regulation activity. The remaining five thought that perhaps the care providers would experience the involvement of the inspectors as a burden and that it could be an effort for them to participate: OA04 “maybe it would cost them too much time” .

### Care providers

The following professions were represented during the focus groups: home care nurses (*n* = 2); district nurse (*n* = 1); general practitioner (*n* = 3); mental health professionals (*n* = 3); pharmacist (*n* = 2); elderly advisor (*n* = 1); and a mental health nurse (*n* = 1).

The involvement of the clients was seen as valuable by the care providers, because their experiences with and opinions about their care were now heard by the inspectors. The care providers noticed that the clients themselves appreciated the conversations with the inspectors. FC02 “what I know from my client who did the interview, that he was very positive about the interview and about the way the inspectors questioned him […] it was correct, friendly and empathetically”. However, according to the care providers the inspectors may have received a biased image of the functioning of the care networks in the region because of the small number of clients who were involved. One important disadvantage of this method was mentioned during both focus groups. This was that the most vulnerable clients were probably not included in the regulatory activity. This was described as follows: FC01 “The clients who are actually most complex, can’t be interviewed, so automatically they will not be selected to be interviewed”. They are, however, an important group who often receive care in their own homes from multiple care providers. The respondents also wondered whether the clients were able to recall information on all the care providers involved since their cognitive functions are often impaired and so many care providers may be involved.

The regulation of care networks was seen as a valuable addition to the current regulation process because it focuses on cooperation between individual care providers. FC01 “It shows loopholes in the cooperation between care providers”. The respondents agreed that they are not always aware of the other care providers involved with a certain client and mentioned that this could be improved. They emphasized, however, that this is not necessary for all clients. The main outcome of the IGJ project for the care providers was that it made them more aware of the lack of cooperation between the different care providers involved. The results did not offer them enough guidance to improve daily practice because they found the presented results were quite general.

The care providers noted that some important means of improving cooperation were lacking in the current system. They expect the IGJ to acknowledge the following aspects when regulating care networks: the involvement of secondary health care providers and the lack of cooperation between the first and secondary care providers; budget cuts; changing money flows resulting in a lack of financing for cooperation; privacy issues surrounding patient records which hinder the exchange of information about a client; competition between care organizations which also hinders full disclosure between care providers. FC01 “Competition between care organizations is difficult, especially when it becomes obligatory to share information, there is not a single commercial party which shares its data.”

The respondents did perceive themselves as part of a care network, but not around one individual client. They all work in a region and they seek cooperation when necessary with the care providers they are familiar with. They are not always aware of the other care providers involved with one particular client. They argue that this is only necessary when there are problems. In those situations, you have to be able to find and contact the right care provider. Some care providers did not agree and mentioned that you always have the responsibility of knowing who is involved: FC02 “I think that as a care provider you have your own responsibilities to assess the care network of a client and to make sure that you take the initiative to contact other care providers involved and to stay in contact. It is then easier to approach them when you need them.”

With regard to how the IGJ project was conducted, the respondents mentioned that they were able to be open and honest about the current situation. However, they expected care providers to be less inclined to give full disclosure to the inspectors when this method is used for regulation with enforcement.

### Inspectors

All seven inspectors involved in the project group participated in the focus group. The clients’ perspective was seen as important since they expected that the clients have the best overview of the care providers involved. The perspective of the client is often different from that of a professional and clients sometimes did not have an opinion on the care they received. FI “A substantial part of the clients did not have an opinion about the care they received, or they mentioned topics that were important to them but were not of interest for the IGJ”. When solely using the perspective of the client, insight into the safety of the delivered care and adherence to professional norms will be lacking. Topics such as kindness and making the time to have a conversation may be important for the client, but are not core elements for the safety of care. The norms used in the framework were not based on the perspective of the client but seen from a professional perspective. The inspectors wondered if they could change the perspective of the norms to better implement the clients’ perspective.

“While using the framework, the inspectors realized that professionals in the care network needed to pay more attention to what extent the clients were able to control their own lives. FI” “an important finding during our project was that it was not the complexity of the care network that is essential [for the functioning of a care network], but the ability of a client to control his or her own life or the involvement of an informal care provider who takes over that control”. A conclusion drawn by the inspectors during the focus group was that the framework should therefore put more emphasis on this matter. The participants in a care network should discuss the control a client has over his or her life, monitor this and jump in when needed since control over one’s life is a continually changing concept.

In its current form, the framework provided information for the inspectors about the functioning of a network around one person, but it lacked the provision of information about both the decision-making process within a care network, and the cooperation between organizations at the regional level. Additional information about cooperation between and within organizations and the local governments is desired from the management and policy level to eventually get a clear picture of how the network functions.

### Logbooks

The researchers analyzed the time invested in the regulatory activity by the inspectors. A distinction was made between direct, indirect and travel time during the whole project. Direct time was seen as conversations, face-to-face or by phone, with the clients or care providers while using the framework. The recruitment of the clients and care providers and preparations for all the conversations were seen as indirect time, as was the analysis of the conversations. Travel time was extensive since the two regions were both remote.

Most time was spent on indirect and travel time, both 41.2% of the total time invested and 17.6% was spent on direct time. Of the indirect time, the three most time-consuming tasks were the analysis of the conversations with the care providers (19.2%), secretarial functions (17.7%) and preparations to recruit the older adults (17.1%).

For the direct time, conversations with the care providers, per client, varied from 20 up to 100 min (mean = 56.83 min). Time spent on conversations with the client varied from 25 to up to 100 min (mean = 93.67 min).

In total, it took about one working week (44 h) to assess the total care network of one client.

## Discussion

We investigated the perceived added value of a newly developed regulatory framework by the IGJ using an explorative qualitative study. Our study shows that in the opinion of the three groups of individuals involved, that is the older adults, professional care providers and inspectors, the framework contributes to the regulatory process of care delivered at home in care networks. The care providers mentioned that the framework identified loopholes in the collaboration between care providers. However, different conditions and barriers were also mentioned. Attention to the ability to control one’s own life, or the situation of the older adult, should be embedded in the regulatory framework better, according to the older adults and the inspectors. Financial and privacy issues hinder the cooperation between care providers and the care providers usually do not perceive themselves as part of a care network around one individual client. Therefore, it can be questioned to what extend care in these situations is actually provided by a network of care providers and how continuity of care is organized. The framework was able to point out these issues, but it also raises questions about the potential for more general application of the regulatory framework. The absence of enforcement during the test likely contributed to the rich information collected about the functioning of a care network. It should be considered carefully in what situations enforcement is applicable. Furthermore, regulating a network around one client took on average 44 h, which is very time consuming for the inspectors.

According to Adams et al. 2005 [29] clients can provide relevant signals about the quality of care, but each situation should be considered carefully on its individual factors when judging its relevance. In our study, involving the client provided the inspectors with the opportunity to explore the situation and the care networks of the selected older adults. For example, it may provide information about whether agreements that have been made between organizations are actually followed through by the care providers. Involving the perspective of the client and their view on the care network was therefore seen as beneficial in our study. However, the respondents stated that the situation of the client has to be considered by the inspectors when involving clients in regulatory activities. They have to be able to provide the inspectors with relevant information, and it should not be a burden for the client. When the client is not able to do this, an informal care provider can provide the relevant information.

Baart (2016) suggests in his essay about quality frameworks that when an inspector talks with the multiple different individuals concerned, this can provide a richer, more valid and legitimate judgment about the quality of care than when they speak to only one individual [40]. In this study different type of respondents were interviewed to assess the quality of care, this contrasts the current majority of regulatory activities where usually checklists are used and not conversations about quality.

The degree of control over one’s life was an important issue that arose from using the framework. The inspectors felt it was important that the coordination of care remained with the client, whenever possible. When the client is able to coordinate his own care, then little interference is required from the professional care providers. However, care providers should assess and monitor the degree of control regularly because it can change quickly. Major life experiences such as the death of a spouse, hospitalization or the onset of diseases like dementia may cause this. Therefore, the inspectors felt that arrangements should be made about which care provider is responsible to monitor this. When the degree of control is monitored, care providers should be able to interfere in time, so the degree of support can be adjusted and the older adults still receive the care they need.

### Barriers

The professional care providers experienced different barriers for collaboration with other care providers involved around a single client, which hinders the functioning of a care network. These barriers were: the involvement of secondary health care providers; budget cuts; changing money flows; a lack of financing for collaboration; and privacy issues for patients. The majority of the barriers they perceived are not new. According to Callister and Wall (2001) and Hall (2005) boundaries experienced in finance, professional expertise and regulations are seen as barriers to deliver seamless services [41, 42]. When considering that these barriers exist for more than a decade, it raises the question whether these barriers can be removed to deliver seamless care.

The care providers indicated that they did not feel they were part of a care network around one individual client at the time of the study. It will probably take time before care providers think and work in terms of networks, also because of the barriers mentioned above. Changes in health care often take time due to the incremental nature of change and the institutional layering in health care [43]. However, it is important to think in terms of care networks to ensure integrated, seamless care because of the increasing number of independently living older adults and the accompanied increasing amount of care delivered in the home setting by different formal and informal care providers.

The transition in long-term care had just started when the framework was being tested. Currently, there are still a lot of changes going on in the field of long-term care and this is not only the case in the Netherlands. Different countries face the challenges of a growing older population. According to Schakel et al. (2016) health care regulation is valuable when it does not restrict the care organizations and encourages them to learn and improve [44]. When using enforcement in health care regulation, it is thought that organizations have fewer opportunities to learn. Whether or not enforcement will be feasible as a next step for the framework depends upon the decision of the IGJ to continue this regulatory activity in a non-exploratory setting.

Different factors influence to what extent the regulatory framework can be applied in daily practice. In traditional situations concerning regulation there is a clear risk, norm and applicant. But these are often unclear when regulating care networks [45]. The involvement of many different formal and informal care providers, the local inspectorate, and the lack of norms regarding the quality and safety in care networks are all complicating factors to consider when regulating care networks.

### Implications

Our findings have implications for the regulatory body using the framework, (in this case the IGJ), care providers, local government and future research. Regulation of care networks by a regulatory body puts working in networks on the agenda of care providers. It would be desirable to communicate a clear idea of what is expected of cooperation and coordination in care networks. However, this change in thinking and working will take time [42]. The regulatory body should keep the barriers to working in care networks mentioned by the care providers in mind when they regulate care networks. It is also desirable that both central and local government take notice of the barriers and boundaries relating to finance and privacy and where possible act on them.

We would recommend considering carefully for which elements of the framework enforcement is appropriate. When enforcement is applied, care providers will face consequences when they do not meet the criteria as set in the framework. More time is needed to experiment and learn from the field. Many changes are still taking place in long-term care and barriers and boundaries to work in care networks around one person are perceived by the care providers. Another argument for this recommendation is that the care providers mentioned that they gave full disclosure of their views and practice because there were no consequences, unless serious dysfunction was established.

To work in care networks, it is essential for care providers to know which care providers are involved. We therefore recommend that care providers assess who is involved when they administer a new client. This may lower the barrier to contact one another and cooperate when necessary. In the past years there is increasing attention for integrated care, especially for vulnerable groups of people. For example, the ‘Development Model for Integrated Care’ has been developed by Minkman et al. [46]. This, however, differs from the care network as discussed in this study since the care networks are not formalized cooperation’s between care providers.

All three types of respondents emphasized that the ability to control one’s own life is an important aspect to take into account. We recommend regulatory bodies to pay more attention to measures taken by care providers to assess this ability of a client. Moreover, the degree to which this concept is constantly changing needs to be considered. According to Proot et al. offering support tailored to the degree of autonomy can stimulate active participation in the care process [47]. Including this element within the framework, may stimulate care providers to become more aware of this. The care providers should have insight into the capability of the client to coordinate their own care. Also, the ability to control one’s own life is highly changeable and should be monitored or checked regularly by informal or formal care providers to support the client in the right way. Who is responsible to monitor this and in what way, depends on the agreements made by the care providers who are involved around that specific client.

An implication for future research is the need to evaluate the possible effects of the regulatory framework for regulating long-term care delivered at home. For example the number of care providers involved who know each other. Also, changes in the cooperation between care providers in a care network can be analyzed when using a baseline and follow up questionnaire among care providers. A second possibility is to analyze changes in the experiences of care coordination as perceived by the care recipient and their informal care provider, using a concept such as client satisfaction.

### Considerations

Research into the regulation of long-term care delivered at home is a relatively new area. This study adds to our knowledge by using several research methods to identify the perceived potential and barriers of a newly developed regulatory framework.

Using a qualitative design provided insight into the respondents opinions about the IGJ framework. A quantitative design would not have allowed the researchers to gain the underlying thoughts and the sample would have been too small. However, all respondents spoke with the researchers, face to face or by phone and this may have contributed to socially desirable answers, even though confidentiality of the answers was guaranteed by the researchers.

The IGJ may have an influence on different parties in the care system, therefore it is valuable for them to reflect on its own functioning. Dahler-Larsen (2011) indicated that when inspectorates reflect on their own functioning they become more humble and proactive than omniscient [48]. This study reflects on work carried out by the IGJ and this may have a positive effect upon the cooperation of field parties and on public opinion about the IGJ.

The most vulnerable clients and the ones avoiding care probably did not participate in the IGJ project. However, the functioning of their care network may be even more important since they probably cannot fully manage and coordinate it themselves. In the future it is important that the IGJ is able to contact the more vulnerable clients and involve their informal care provider in order to assess their care network and experiences compared with the care received.

## Conclusions

This study investigated the perceived added value of, and barriers to a newly developed regulatory framework for healthcare networks by the Dutch Healthcare Inspectorate. Internationally, the development of healthcare regulation on care networks is a challenge. Our study drew on the experiences of the older adults involved, professional care providers and the inspectors. Our results show that evaluating the development of new regulatory activities improves the quality and shows boundaries of the applicability of these activities. Two important aspects of the framework were innovative. These were starting from the experiences of the client and mapping the care network from the clients’ perspective. These two aspects were seen as valuable by all respondents.

An important barrier to applying the framework is the time consuming aspect. The question is in which situations this time investment is justified. The framework is valuable when the regulator wants to explore new and unknown areas, such as care networks. Also, it can have added value in situations where the risks are unknown or in response to signals about insufficient cooperation in the care for older adults living independently. However, the investment in time required, and the limited reach when investigating individual care networks, make this framework less appropriate as a standard method for regulation. We recommend to regulatory bodies to take a critical view in which situations the framework can be applied and whether enforcement is applicable in that particular situation. The framework can provide rich information about the actual situation and is meaningful to apply when exploring care networks as a new area.

The results of this study imply some adjustments are required to the framework. All respondents perceived the ability of a client to control one’s own life as important. We recommend paying more attention in the framework to this topic and checking whether care providers assess this ability of a client. The professional care providers also experienced financial and privacy barriers both to cooperation and to thinking in terms of a care network. These barriers should be considered while using the framework [49]. However, regulating care networks stimulates cooperation between care providers.

## Abbreviations

AWT: Academic Collaborative Center on Supervision
IGJ: Dutch Health and Youth Care Inspectorate
TFI: Tilburg Frailty Indicator
VU(mc): Vrije Universiteit (Medical Center)
VWS: Dutch Ministry of Health, Welfare and Sport
Wlz: Long Term Care Act
Wmo: Social Support Act
Zvw: Health Insurance Act
